# Lymphogranuloma venereum is on the rise in Belgium among HIV negative men who have sex with men: surveillance data from 2011 until the end of June 2017

**DOI:** 10.1186/s12879-018-3600-0

**Published:** 2018-12-20

**Authors:** Irith De Baetselier, Achilleas Tsoumanis, Ruth Verbrugge, Bénédicte De Deken, Hilde Smet, Saïd Abdellati, Vicky Cuylaerts, Ludwig Apers, Tania Crucitti

**Affiliations:** 1 Department of Clinical Sciences, Institute of Tropical Medicine, Nationalestraat 155, 2000 Antwerp, Belgium; 2 Epidemiology of Infectious Diseases, Scientific Institute of Public Health, Juliette Wytsmanstraat 14, 1050 Brussels, Belgium

**Keywords:** Lymphogranuloma venereum, LGV, Chlamydia trachomatis, Pre-exposure prophylaxis, Men who have sex with men, MSM

## Abstract

**Background:**

The number of cases of Lymphogranuloma venereum (LGV) is increasing in Europe. The described epidemic is mostly confined to HIV positive men who have sex with men (MSM). However, dissemination of LGV from HIV positive to HIV negative MSM could take place due to the implementation of pre-exposure prophylaxis (PrEP) and subsequent possible decrease in condom use. We describe here the LGV epidemiology in Belgium before the PrEP-era, starting from 2011 up to the end of the first half of 2017.

**Methods:**

A descriptive analysis of the socio-demographic and clinical characteristics of all LGV cases was performed. Fisher’s exact test was used to compare symptomatic to asymptomatic patients. Logistic regression models were used to check for trends over time for: number of LGV cases, HIV status and symptoms.

**Results:**

The number of LGV cases rose by a factor four, from 21 in 2011 to 88 in 2016, and regression models showed a positive trend estimate of 14% increase per half year (*p* < 0.001). LGV decreased among HIV positive cases (odds ratio (OR): 0.79, p < 0.001) and increased among HIV negative cases (OR: 1.27, *p* < 0.001). In addition, a rise in the number of asymptomatic LGV cases (6.7%) was observed (OR:1.39, *p* = 0.047). Asymptomatic cases were also less likely to be HIV (*p* = 0.046) or Hepatitis C positive (*p* = 0.027).

**Conclusions:**

The rise of LGV in HIV negative MSM has now been documented. If we aim to halt the epidemic in HIV negative MSM, future public health strategies should include LGV testing of all *Chlamydia trachomatis* positive samples from MSM.

## Background

Lymphogranuloma venereum (LGV) is a bacterial sexually transmitted infection (STI) caused by the L serovars of *Chlamydia trachomatis* (CT). Although initially a tropical infection characterized by inguinal buboes, LGV under the form of proctocolitis has become endemic among men who have sex with men in Europe. The CT L serovar is more invasive compared to the other serovar types leading to more severe sequelae and has been suggested to be associated with increased HIV and other STI infection [1]. The number of infections is increasing in many Western and Central European countries where enhanced LGV surveillance has been implemented. In fact, the number of European cases reported in 2014 increased by 32% compared to 2013 [2]. However, the increase is probably an underestimate as not all countries have the capacity to identify CT serovar/genotype L and to report LGV. In addition, only suspected LGV cases are tested and recent reports have shown that asymptomatic infections may be higher than initially thought [3–5].

The LGV epidemic is mostly confined to HIV positive men who have sex with men (MSM) [6]. The HIV prevention landscape has changed impressively over the past few years due to the implementation of treatment as prevention and pre-exposure prophylaxis (PrEP). In Belgium, PrEP has been reimbursed since June 2017 [7]. PrEP in Belgium can only be prescribed by an HIV specialist working in an Aids Reference Centre such as the HIV/STI clinic at the Institute of Tropical Medicine (ITM). According to PrEP guidelines, STIs need to be monitored at least six monthly. Previously, urine sampling was performed for STI screening on a routine basis, however, European guidelines for LGV recommend that MSM who report receptive anal sexual practices in the previous 6 months, are screened twice a year for anorectal CT infection [8]. This recommended biannual STI screening may detect asymptomatic STI infections more frequently and thus prevent further transmission [9].

To explore differences in behavioral and clinical characteristics of LGV cases over time, we describe here the LGV epidemiology in Belgium including the positivity rate of LGV cases among men attending an Antwerp HIV/STI clinic for the pre-PrEP era, starting from 2011 up to and including the first half of 2017. Repeater- and asymptomatic infections were reviewed in depth.

## Methods

### National surveillance of LGV

In 2011, the ITM was recognized as the National Reference Centre for STIs (hereafter called NRC) and has since then implemented the laboratory surveillance of LGV. Physicians or microbiologists suspecting a case of LGV are urged to send the patient’s sample with accompanying socio-demographic and clinical data to the NRC for confirmation of CT genotype L. In addition, health care providers are instructed to send CT positive samples of patients at risk for LGV to the NRC regardless the presence of symptoms.

All biological material (i.e., anorectal-, pharyngeal-, ulcer dry swabs or urine samples) is tested for CT using the Abbott Real-Time CT/NG PCR according to manufacturer’s instructions. When testing positive for CT, a confirmation in-house real-time PCR using previously published *pmpH* gene primers/probes is performed to differentiate LGV (L genotypes) from non-LGV (A-K genotypes) strains [10]. In the case of DNA extracts, only the confirmation LGV/non-LGV real-time PCR is performed.

All data from LGV confirmed cases are coded and submitted on a regular basis to the Belgian Institute of Public Health through a secured web portal.

Duplicate records of all LGV infections found by the NRC are removed, based on date of birth, date of consultation, postal code and gender. An LGV re-infection is defined as a separate episode when a new infection is recorded for the same patient at least 3 months after the first episode, to exclude potential treatment failures [11].

### Routine CT detection at the HIV/STI clinic, ITM

Besides being the NRC, the ITM has a large HIV/STI clinic and followed close to 3000 HIV positive patients at the end of 2016. In addition, the HIV/STI clinic is also home to a low threshold clinic that offers free-of-charge screening for HIV and STIs in asymptomatic individuals. In 2016, 2307 individuals, mainly belonging to high risk groups (MSM, transgenders and anyone who engages in unsafe sex), made use of this service. For sexually active MSM, screening for STIs in urine and HIV testing is performed at least twice yearly. In the case of symptomatic individuals, samples are collected from the suspected infection site. The number of CT analyses requested by the HIV/STI clinic from 2011 till 01 July 2017 was retrieved. Due to the fact that no female LGV cases were detected in the clinic, female samples were excluded from the analysis. Results that were invalid due to inhibition, wrong or insufficient volume of sample or results that could not be confirmed by the LGV/non-LGV real-time PCR were removed from the dataset before analysis.

### Statistical analysis

The descriptive analysis was performed using IBM SPSS Statistics version 24. Continuous variables are summarized as medians and interquartile ranges (IQR) and categorical ones as counts and percentages. The longitudinal analysis was performed using R version 3.4.1 [12]. Poisson regression models were fitted to the aggregated LGV data by year and by six-monthly period, with the number of cases as the outcome and the respective time variable as the only covariate. Logistic regression models were fitted to the individual data for all the other variables of interest. In all models, time was treated as numerical. The trend estimates are presented as rates for the Poisson models and as odds ratios (ORs) for the logistic regression models with 95% confidence intervals (CIs).

Furthermore, Fisher’s exact test was used to compare variables of interest between asymptomatic and symptomatic LGV cases. All tests were two-sided and significance level was set at 5%.

## Results

### Overall Belgian LGV epidemic

A total of 343 LGV cases were identified by the NRC, of those 186 were from the HIV/STI clinic and 157/765 cases were provided by peripheral laboratories The socio-demographic and clinical data of the 343 cases reported between 2011 and the end of June 2017 are compiled in Table 1. Percentages were calculated excluding missing data. The mean age was 40.6 years (IQR: 33–48) and LGV episodes were detected in 297 men, two transwomen and one woman. Most of the cases originated from patients living in the Flemish region of Belgium (75%). Of those with LGV for whom data were available, almost all were identified as MSM (98.7%). Three heterosexual transmissions were reported among men (1.2%) albeit that they had rectal LGV infections; two of them were HIV positive and all suffered from symptoms, suggestive of proctitis. The HIV status was known in 90.0% (308/343) of the cases whereby 84.7% (261/308) were HIV positive and 15.3% (57/308) HIV negative. Of the HIV positive men, 73.9% (193/261) were MSM.

**Table 1:** Socio-demographic and clinical data of LGV cases starting from 2011 until end of June 2017

	2011 n(%)	2012 n(%)	2013 n(%)	2014 n(%)	2015 n(%)	2016 n(%)	S1 2017 n(%)	TOTAL
Number of LGV cases	21	23	45	58	62	88	46	343
Gender	343/343 known							
Male or Trans female	21 (100%)	23 (100%)	45 (100%)	58 (100%)	61 (98%)	88 (100%)	46 (100%)	342 (99,7%)
Female	0 (0%)	0 (0%)	0 (0%)	0 (0%)	1 (2%)	0 (0%)	0 (0%)	1 (0,3%)
Transmission of all men	241/343 known							
MSM	13 (100%)	13 (100%)	26 (100%)	42 (100%)	52 (98%)	55 (98%)	37 (97%)	238 (99%)
HETERO	0 (0%)	0 (0%)	0 (0%)	0 (0%)	1 (2%)	1 (2%)	1 (3%)	3 (1%)
Age scale	343/343 known							
20–30	3 (14%)	5(22%)	2 (4%)	10 (17%)	17 (27%)	14 (16%)	6 (13%)	57 (17%)
31–40	7 (33%)	7 (30%)	17 (38%)	28 (48%)	14 (23%)	27 (31%)	17 (37%)	117 (34%)
41–50	8 (38%)	8 (35%)	21 (47%)	15 (26%)	17 (27%)	27 (31%)	14 (30%)	110 (32%)
51–60	2 (10%)	3 (13%)	5 (11%)	4 (7%)	14 (23%)	18 (20%)	8 (17%)	54 (16%)
> 61	1 (5%)	0 (0%)	0 (0%)	1 (2%)	0 (0%)	2 (2%)	1 (2%)	5 (1%)
Geographical location in Belgium	338/343 known							
Living in Flemish region	17 (81%)	20 (91%)	37 (84%)	36 (63%)	47 (76%)	61 (69%)	36 (81%)	254 (75%)
Living in French region	2 (10%)	1 (5%)	2 (5%)	3 (5%)	7 (11%)	11 (12%)	3 (7%)	29 (9%)
Living in the capital region	2 (10%)	1 (5%)	5 (11%)	18 (32%)	8 (13%)	16 (18%)	5 (11%)	55 (16%)
Kind of Sample	337/343 known							
Anorectal	17 (85%)	22 (96%)	37 (86%)	51 (89%)	56 (92%)	84 (95%)	40 (89%)	307 (91%)
Genital	3 (15%)	1 (4%)	3 (7%)	6 (11%)	2 (3%)	3 (3%)	2 (4%)	20 (6%)
Urine	0 (0%)	0 (0%)	2 (5%)	0 (0%)	3 (5%)	1 (1%)	1 (2%)	7 (2%)
Inguinal	0 (0%)	0 (0%)	1 (2%)	0 (0%)	0 (0%)	0 (0%)	1 (2%)	2 (1%)
Eye fluid	0 (0%)	0 (0%)	0 (0%)	0 (0%)	0 (0%)	0 (0%)	1 (2%)	1 (0,3%)
HIV Status	308/343 known							
Positive	18 (100%)	20 (100%)	40 (95%)	45 (88%)	45 (75%)	63 (84%)	30 (71%)	261 (85%)
Negative	0 (0%)	0 (0%)	2 (5%)	6 (12%)	15 (25%)	12 (16%)	12 (29%)	47 (15%)
Symptoms	232/343 known							
Proctitis	6 (75%)	2 (50%)	19 (63%)	40 (89%)	43 (81%)	41 (76%)	25 (66%)	176 (76%)
Inguinal lymphadenopathy or ulcer	0 (0%)	1 (25%)	3 (10%)	0 (0%)	3 (6%)	2 (4%)	3 (8%)	12 (5%)
Other abdominal symptoms^a^	0 (0%)	0 (0%)	3 (10%)	3 (7%)	3 (6%)	0 (0%)	1 (3%)	10 (4%)
Genital Ulcer	2 (25%)	0 (0%)	1 (3%)	1 (2%)	0 (0%)	1 (2%)	1 (3%)	6 (3%)
Urethritis	0 (0%)	0 (0%)	1 (3%)	1 (2%)	0 (0%)	0 (0%)	0 (0%)	2 (1%)
Other symptoms^b^	0 (0%)	0 (0%)	0 (0%)	0 (0%)	0 (0%)	1 (2%)	2 (5%)	3 (1%)
Asymptomatic	0 (0%)	1 (25%)	3 (10%)	0 (0%)	4 (8%)	9 (17%)	6 (16%)	23 (10%)
Co-infections other than HIV	197/343 known							
None	6 (40%)	0 (0%)	2 (9%)	7 (25%)	17 (40%)	20 (37%)	10 (33%)	62 (31%)
Only one	6 (40%)	5 (83%)	20 (91%)	21 (75%)	24 (57%)	34 (63%)	15 (50%)	125 (63%)
Two or more	3 (20%)	1 (17%)	0 (0%)	0 (0%)	1 (2%)	0 (0%)	5 (17%)	10 (5%)
Gonorrhea	4 (24%)	5 (71%)	11 (41%)	13 (41%)	9 (19%)	17 (28%)	10 (28%)	69 (35%)
Syphilis	2 (12%)	2 (29%)	9 (33%)	4 (13%)	8 (17%)	9 (15%)	6 (17%)	40 (20%)
Hepatitis C	2 (12%)	0 (0%)	4 (15%)	6 (19%)	7 (15%)	10 (17%)	0 (0%)	29 (15%)
Chlamydia A-K	0 (0%)	0 (0%)	0 (0%)	1 (3%)	4 (9%)	0 (0%)	4 (11%)	9 (5%)
Hepatitis B	2 (12%)	0 (0%)	1 (4%)	1 (3%)	0 (0%)	1 (2%)	2 (6%)	7 (4%)
Genital herpes	0 (0%)	0 (0%)	0 (0%)	0 (0%)	2 (4%)	0 (0%)	1 (3%)	3 (2%)
Other^c^	1 (6%)	0 (0%)	0 (0%)	0 (0%)	0 (0%)	3 (5%)	3 (8%)	7 (4%)

% removing unknowns – deviations from 100% can be due to rounding

*S* Six-monthly period

^a^other abdominal symptoms include rectal ulcers or lesions, fistulas, diarrhoea, constipation, peri-anal pain

^b^other symptoms include conjunctivitis, arthritis or unknown other symptoms

^c^other co-infections include shigella, campylobacter, varicella zoster, *Mycoplasma genitalium*

Of the samples with known biological origin (337), the majority were anorectal (91.1% - 307/337), followed by genital samples (5.9% - 20/337) and urine (2.1% - 7/337). Two LGV infections were detected in samples of inguinal glands (0.6%) and one swab from eye material was also found to be LGV positive (0.3%). The most reported symptom was proctitis (75.9% - 176/232), followed by inguinal lymphadenopathy or the presence of an inguinal ulcer (5.2% - 12/343). Abdominal symptoms such as diarrhoea, constipation, rectal ulcers, lesions or fistulas and peri-anal pain were reported in 4.3% of the cases (10/232). Other symptoms were genital ulcers (2.6% - 6/232), urethritis (0.9% - 2/232), arthritis (0.4% -1/232) and conjunctivitis (0.4% - 1/232). In 23 cases, LGV infection was found to be asymptomatic (9.9%).

For almost 70% of the LGV patients one (63.5% - 125/197) or more (5.1% - 10/197) co-infections other than HIV were reported. Gonorrhea was most frequently reported (35.0% - 69/197) followed by syphilis (20.3% - 40/197) and hepatitis C (HCV) (14.7% - 29/197). Chlamydia genotypes A-K, hepatitis B, genital herpes, *Mycoplasma genitalium*, Shigella, Campylobacter and Human Papilloma Virus were reported less frequently (< 5%) and co-infections were absent in 31.5% of the cases (62/197).

### Longitudinal data of the LGV epidemic

**Fig. 1:**
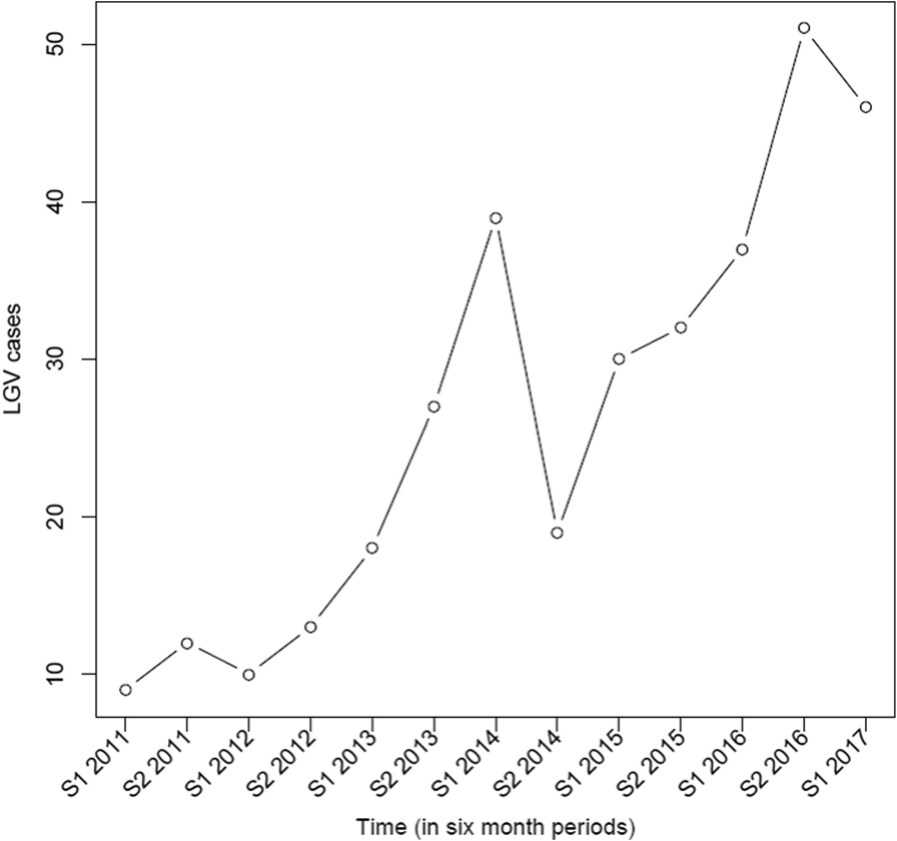
Number of LGV cases over time in Belgium from 2011-S1 2017. S= six months

The number of cases rose by a factor four, from 21 in 2011 to 88 in 2016 (see Fig. 1). In the first half of 2017, 46 cases were identified. Poisson regression models showed a positive trend estimate of 14% increase per half year (95%CI: 1.11–1.17) (*p* < 0.001). Surprisingly, in the second half of 2014, a drop in LGV cases was noted, without any plausible explanation. Piece-wise regression was used to segment the regression in the first half of 2014 and showed that the positive trend estimate rate increased to 30% per 6 month period (95% CI: 1.19–1.43) (*p* < 0.001) for either period before and after the break.

**Fig. 2:**
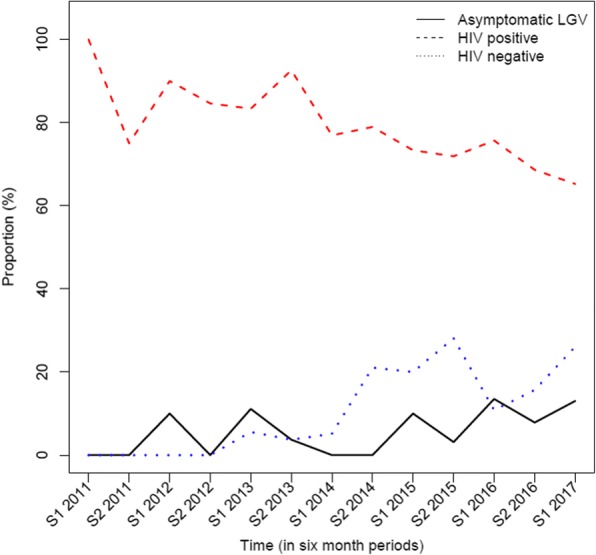
Proportion of HIV-positive, HIV-negative and asymptomatic LGV cases over time. S= six months

Patient characteristics over the years with regard to sexual behaviour and demographics were relatively stable over the years. Figure 2 shows the percentage of HIV positive, HIV negative and asymptomatic cases over time. Regression analyses showed that the number of asymptomatic cases increased borderline significantly per year (*p* = 0.047) with an OR of 1.39 (95% CI: 1.02–1.97). When breaking down the time line per 6 month period, the OR decreased to 1.16 (95% CI: 0.99–1.39), with a *p*-value of 0.06. A decrease in HIV positive cases (OR 0.79 (95% CI: 0.69–0.88)) and an increase in HIV negative cases (OR 1.27 (95% CI: 1.13–1.45)) over time was, however, highly statistically significant (*p* < 0.001). The rates of gonorrhea or syphilis co-infections did not change significantly over time (*p* = 0.12 and 0.32, respectively) and are not presented in the figure.

### Asymptomatic cases

Over the time period, 23 asymptomatic cases were reported: 22 men (all MSM) and one woman (Table 2). The vast majority of asymptomatic LGV infections were anorectal; only two urinary infections were reported (one male, one female).

**Table 2:** Socio-demographic and clinical data of asymptomatic cases starting from 2011 until end of June 2017

	2011 n(%)	2012 n(%)	2013 n(%)	2014 n(%)	2015 n(%)	2016 n(%)	S1 2017 n(%)	TOTAL
Number of asymptomatic LGV cases	0	1	3	0	4	9	6	23
Gender	23/23 known							
Male or Trans female	0	1	3	0	3	9	6	22 (96%)
Female	0	0	0	0	1	0	0	1 (4%)
Transmission of all men	22/22 known^a^							
MSM	0	1	3	0	3	9	6	22 (100%)
HETERO	0	0	0	0	0	0	0	0 (0%)
Age scale	22/22 known^a^							
31–40	0	1	1	0	0	4	3	9 (41%)
41–50	0	0	2	0	1	4	3	10 (45%)
51–60	0	0	0	0	2	1	0	3 (14%)
Geographical location in Belgium	22/22 known^a^							
Living in Flemish region	0	1	2	0	3	7	5	18 (82%)
Living in French region	0	0	1	0	0	1	1	3 (14%)
Living in the capital region	0	0	0	0	0	1	0	1 (4%)
Kind of Sample	22/22 known^a^							
Anorectal	0	1	3	0	3	8	6	21 (95%)
Urine	0	0	0	0	1	0	0	1 (5%)
HIV Status	22/22 known^a^							
Positive	0	1	3	0	2	5	3	14 (64%)
Negative	0	0	0	0	1	4	3	8 (36%)
Co-infections other than HIV	21/22 known^a^							
None	0	0	0	0	2	1	2	5 (24%)
Only one	0	0	2	0	1	5	2	10 (48%)
Two or more	0	1	1	0	0	3	1	6 (29%)
Gonorrhea	0	1	2	0	0	6	1	10 (48%)
Syphilis	0	1	2	0	0	2	1	6 (29%)
Chlamydia A-K	0	0	0	0	1	0	1	2 (10%)
Hepatitis B	0	0	0	0	0	1	0	1 (5%)
*Mycoplasma genitalium*	0	0	0	0	0	2	1	3 (14%)

% removing unknowns – deviations from 100% can be due to rounding

*S* Six-monthly period

^a^only male cases are presented here

In total, 6.7% (23/343) of all LGV cases were found to be asymptomatic, of those with available data, this percentage increased to 9.7% (21/217) for rectal LGV infections. Seventeen percent (8/47) of the documented HIV negative LGV cases were asymptomatic.

Statistical analysis with Fisher’s Exact test revealed that asymptomatic patients were borderline less likely to be HIV positive (*p* = 0.046) and were less likely to have HCV co-infection (*p* = 0.027). Other parameters such as gonorrhea or syphilis co-infection were not significant (*p* = 0.144 and 0.388, respectively).

### Re-infections

During this time period, 300 patients were diagnosed with LGV. Of them, 28 were identified as having repeated infections (9.3%).

Time between repeated infections varied from 4 months to 4 years. Most of them (17) were infected twice (4 to 53 months apart), eight reported three episodes, two patients had an LGV infection for the fourth time during this period, and one had five LGV infections (MSM, HIV positive, infection period age range: 35–41 years). The median time to the second episode was 20 months with a range of 4–53 months and 42.9% (12/28) of the repeaters had a second episode within less than 12 months. HIV positivity was confirmed in 25 men (89.3%). Two remained HIV negative and the HIV status is unknown for one patient (all had two episodes within 5 to 15 months). Of the 71 LGV infections found in repeaters, 37 had an STI co-infection (52.1%).

### CT epidemic at the HIV/STI clinic

**Fig. 3:**
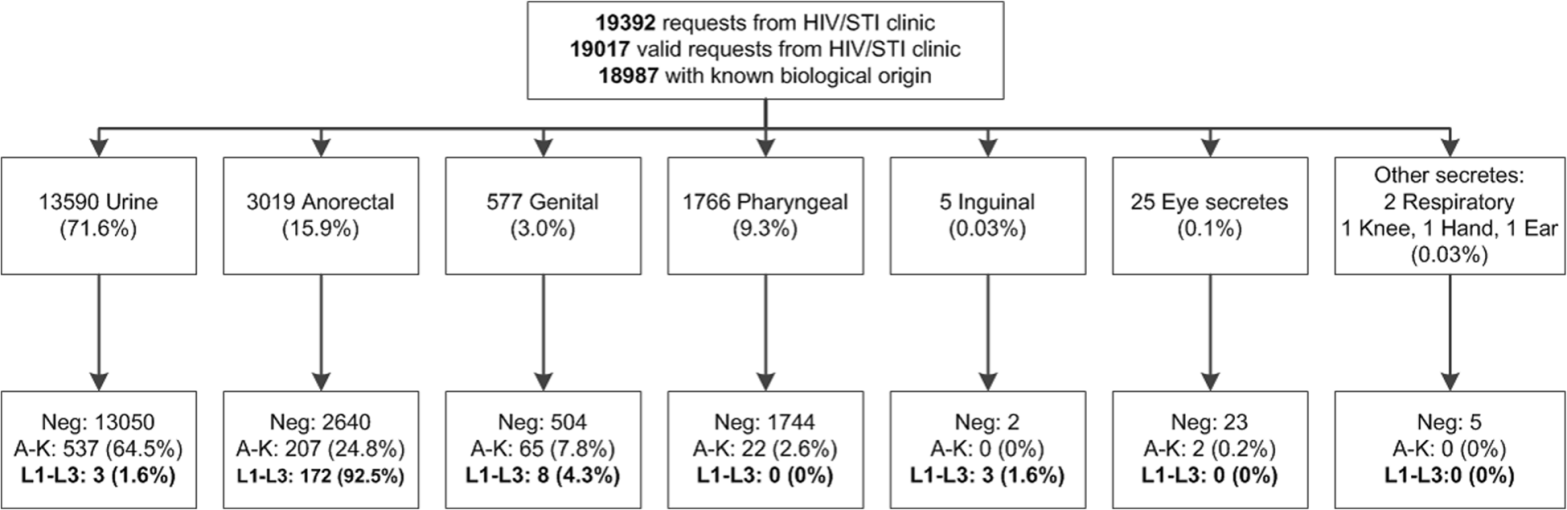
Results of CT testing in men at the HIV/STI clinic of ITM from 2011-S1 2017. S = a six month period

A total of 19,017 valid requests for CT analysis among male patients were received from the HIV/STI clinic including the low threshold centre (Fig. 3). In all, CT was detected in 1019 /19017 cases (5.4%). Genotypes A-K and L were detected in 833 cases (4.4%) and in 186 cases (1.0%), respectively. LGV was the most prevalent in rectal samples (92.5%), followed by genital samples (4.3%). No pharyngeal LGV was detected.

**Fig. 4:**
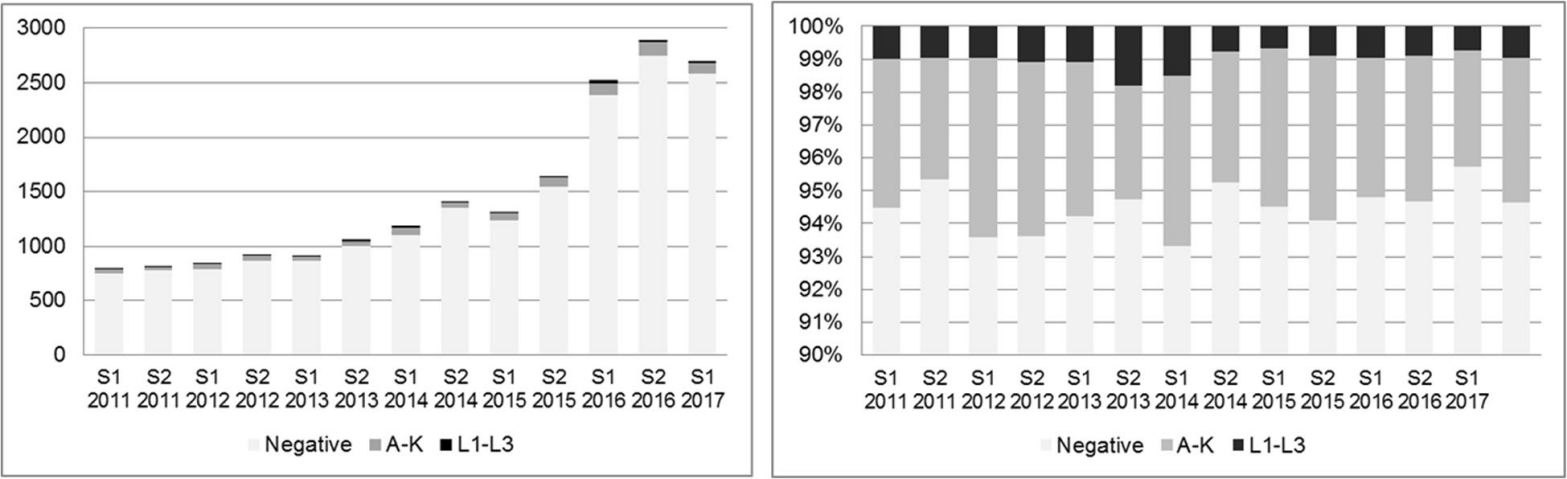
Number of CT analyses among men at the HIV/STI clinic including results over time. The graph on the right hand side represents positivity (%) of A-K and L-types among the samples over time presented in six month periods

The number of CT analyses is depicted in Fig. 4 and shows an large expansion in the number of test starting in 2015, however the positivity rate for Chlamydia remained the same over the 6 month periods and fluctuated between 4.7–6.7%. The LGV positivity rate did not increase over the months and remained around 1.0% (except for S2 2013 with a peak of 1.8%) (Fig. 4).

## Discussion

The number of LGV cases is rising in Belgium, as well as in neighbouring countries [2]. This upward trend might have been influenced by the fact that the Belgian government promoted awareness of LGV among health care providers and microbiologists [13]. Indeed, at the ITM’s HIV/STI clinic, the number of requests per half year rose above 1000 during the second half of 2013 and reaches now 2500 requests every 6 months. Ongoing HIV prevention projects with frequent STI screening among high risk populations may have contributed to this increasing testing trend. However, despite enhanced testing the prevalence of LGV at the HIV/STI clinic remained stable during the years we studied and fluctuated at around 1.0%.

The typical LGV patient (HIV positive MSM presenting with proctitis or other abdominal symptoms) was found among 118 men (39.5%) accounting for 135 LGV cases [14]. Hereby rectal LGV (307/337–91.1%) forms the vast majority of the cases, which is in line with previously described epidemics [4, 15]. The mode of transmission of LGV has been questioned and possible urethral and pharyngeal silent reservoirs have been suggested, however, the evidence is sparse [4, 16–18]. Being one of the few laboratories that differentiates all positive CT on a routine basis, we had hoped to identify the hidden link in the epidemic. However, none of the pharyngeal samples tested positive for LGV and only a few genital LGV infections were detected (8 out of 577 genital samples). Intriguingly, one case of LGV conjunctivitis was detected in the first half of 2017.

During the reported period, 28 patients had repeated infections (9.3%) which is in line with previous reports [4, 11]. Out of these, 26 were HIV positive MSM and co-infections were described in 37 episodes. More worrisome is the observation that three repeaters with gonorrhea as co-infection were asymptomatic. It has been speculated that the central position of LGV repeaters in the sexual network, together with the asymptomatic carriers, may be an important factor in the LGV epidemic [11, 16]. Frequent STI screening (eg. quarterly) among those individuals is therefore recommended.

LGV infection in women was found to be rare [19–21]. Only one woman with LGV was detected over the 6 year period in Belgium and she was screened because her partner was a sexual contact of an LGV case. In addition to this female asymptomatic case, 22 other asymptomatic cases were reported, all among MSM. Fourteen were HIV positive, the other eight were HIV negative. Recent publications show that asymptomatic cases are frequently detected [3–5]. A German study even showed that 42% of 19 documented LGV infections were asymptomatic, but the low number of LGV infections reported in that study limits conclusive interpretations. In that limited study, eight LGV infections were found in HIV negative men, of whom five did not present symptoms (62.5%). We could not detect such a high number of asymptomatic cases (9.9%) which could imply that the Belgian LGV epidemic is larger than herein described. The number of asymptomatic LGV cases is, however, rising and this increase over time warrants attention. We showed that asymptomatic cases are more likely to be HIV negative (borderline *p* = 0.046) and HCV negative (*p* = 0.027). Almost three quarters (72.7%) of the asymptomatic carriers manifested another STI. It is now well documented that LGV may increase the risk for acquisition and transmission of other STIs, including HIV and HCV [22, 23]. Within the group of HIV negative men, we observed a significant rise of LGV (*p* < 0.001). This finding indicates that the spread of LGV is no longer as confined to sexual networks of HIV positive MSM, and screening of high risk HIV negative MSM is therefore warranted.

### Availability of PrEP

Due to the availability of PrEP, changes in sexual behaviour could take place. For example, a number of HIV seropositive partners have stated that they would be comfortable engaging in condomless anal sex if their partner was on PrEP, and individuals on PrEP have also experimented with anal receptive intercourse instead of being exclusively anally insertive [24, 25]. In fact, the emergence of HCV in high-risk HIV negative MSM using PrEP has also been described [26, 27]. The availability of PrEP might therefore initiate the dissemination of LGV from HIV-positive to HIV-negative MSM.

LGV is not a notifiable disease in Belgium, nevertheless, national surveillance is in place for LGV. The NRC is one of the scarce laboratories, and possibly also the only one in Belgium, able to differentiate LGV from non-LGV strains. Consequently, asymptomatic patients will be treated for non-LGV CT infection in other Belgian settings, yet suboptimal treatment, e.g. a one-week course of doxycycline instead of a three-week course, may not prevent onward transmission [3, 28, 29]. We therefore advocate that all CT positive samples from PrEP users, including those who request PrEP, are referred to the NRC for genotype differentiation and LGV confirmation.

### Limitations and weaknesses

Our study has several limitations: First, a major weakness of this investigation was that some of the behavioural parameters were missing. We relied entirely on the information provided by the physicians or microbiologists. The request form documenting the necessary information from LGV patients at the ITM’s HIV/STI clinic was not rechecked with the clinic notes for correctness. Second, most of the LGV cases we present here come from the Flemish region of Belgium. Indeed, the HIV/STI clinic is located in Antwerp, which may have introduced a bias regarding the geographical distribution of the LGV cases. Third, the NRC started with the confirmation of LGV cases in 2011, so the increase over the years could be due to the increased awareness of health care providers but also to ongoing HIV prevention projects at the HIV/STI clinic. However, the rise in HIV negative LGV cases cannot only be attributed to these projects, as cases from HIV negative individuals were also received from other laboratories. Fourth, our sample size over the years is still small and this may have introduced a bias in the regression analyses. Finally, we have not yet performed sequence typing to document the genetic diversity of the LGV cases over recent years because of budgetary constraints. We do, however, advocate that sequence typing should be performed on all samples from the past 2 years to document further the epidemic in Belgium. In addition, discrimination between the L-genotypes could shed more light on why some infections remain asymptomatic.

## Conclusions

To conclude, LGV infection is a serious concern for the MSM community in Europe. If we aim to halt the epidemic in HIV negative MSM, future public health strategies should include testing for LGV on all CT positive samples from MSM, irrespective of their HIV status, so that they, and their partners, can be treated correctly.

## Abbreviations

CI: Confidence intervals
CT: Chlamydia trachomatis
HCV: Hepatitis C Virus
HIV: Human immunodeficiency virus
IQR: Interquartile range
LGV: Lymphogranuloma venereum
MSM: Men who have sex with men
NRC: National Reference Center
OR: Odds ratio
PrEP: Pre-exposure Prophylaxis
STI: Sexually transmitted infection
